# Attosecond betatron radiation pulse train

**DOI:** 10.1038/s41598-020-72053-z

**Published:** 2020-09-15

**Authors:** Vojtěch Horný, Miroslav Krůs, Wenchao Yan, Tünde Fülöp

**Affiliations:** 1 Department of Physics, Chalmers University of Technology, 412 96 Gothenburg, Sweden; 2 Institute of Plasma Physics, Czech Academy of Sciences, Za Slovankou 1782/3, 182 00 Praha 8, Czech Republic; 3 Institute of Physics, Czech Academy of Sciences, ELI BEAMLINES, Na Slovance 1999/2, 182 21 Praha 8, Czech Republic; 4 Key Laboratory for Laser Plasmas (MOE), School of Physics and Astronomy, Shanghai Jiao Tong University, Shanghai, 200240 China

**Keywords:** Laser-produced plasmas, Plasma-based accelerators

## Abstract

High-intensity X-ray sources are essential diagnostic tools for science, technology and medicine. Such X-ray sources can be produced in laser-plasma accelerators, where electrons emit short-wavelength radiation due to their betatron oscillations in the plasma wake of a laser pulse. Contemporary available betatron radiation X-ray sources can deliver a collimated X-ray pulse of duration on the order of several femtoseconds from a source size of the order of several micrometres. In this paper we demonstrate, through particle-in-cell simulations, that the temporal resolution of such a source can be enhanced by an order of magnitude by a spatial modulation of the emitting relativistic electron bunch. The modulation is achieved by the interaction of the that electron bunch with a co-propagating laser beam which results in the generation of a train of equidistant sub-femtosecond X-ray pulses. The distance between the single pulses of a train is tuned by the wavelength of the modulation laser pulse. The modelled experimental setup is achievable with current technologies. Potential applications include stroboscopic sampling of ultrafast fundamental processes.

## Introduction

Sub-femtosecond high brightness X-ray pulses are in high demand by research communities in the fields of biology, material science or femtochemistry^1^, as well as by industry and medicine^2^. Such pulses can be used as a diagnostic tool to resolve the structure and dynamics of dense matter, proteins, and study fundamental physical phenomena such as chemical reactions, lattice vibrations or phase transitions. Currently, high brightness X-ray sources are produced by large scale facilities based on radiation emission by relativistic electron bunches, e.g. synchrotron light sources^3^ and X-ray free electron lasers^4^. This limits their general availability for many of the potential users. Here, we propose a new method to produce a train of equidistant sub-femtosecond X-ray pulses with a currently available laser systems.

Acceleration of electron bunches by the plasma wakefield driven by laser^5,6^, electron^7^, or proton^8^ beams provides a promising alternative to the aforementioned concepts. The major advantage of plasma based accelerators is their ability to sustain acceleration gradients of the order of hundreds of GeV/m, which is approximately three orders of magnitude higher than is attainable with standard radiofrequency accelerators. Thus, the electrons can be accelerated to energies of the order of hundreds of MeV in a few millimeters. During the acceleration process, the electron bunch undergoes transverse betatron oscillations due to the presence of the transverse electric field. As a result, betatron radiation^9–11^ with a synchrotron-like^12^ spectrum, typically in the X-ray range, is emitted.

The betatron radiation characteristics depend on the electron Lorentz factor $\gamma$, plasma electron density $n_e$, betatron oscillation amplitude $r_\beta$, and number of oscillation periods $N_0$. The radiation spectrum is characterized by a critical energy, close to the peak of the synchrotron spectrum, given in practical units $\hbar \omega _c \text { (eV)} = 5.24\times 10^{-21} \gamma ^2 n_e (\text {cm}^{-3}) r_\beta (\upmu \text {m})$. The average photon number with energy $\hbar \omega _c$ emitted by an electron is $N_X = 5.6\times 10^{-3} N_0K$, where $K= 1.33 \times 10^{-10} \gamma ^{1/2} n_e^{1/2} (\text {cm}^{-3}) r_\beta (\upmu \text {m})$ is the strength parameter^13,14^. Several applications of such betatron sources have been demonstrated, e.g. diagnosing biological samples^15^ and probing extreme states of matter^16^, but others would require higher photon number and benefit from increased energy efficiency and better tunability.

Several recent studies suggest methods for enhancing betatron radiation emission, mostly based on the increase of the betatron oscillation amplitude. This can be achieved by an axial magnetic field, either self-generated or external^17,18^; by a delayed modulation laser pulse^19^; by the interaction of the electron beam with a high intensity optical lattice formed by the superposition of two transverse laser pulses^20^; by using structured laser pulses^21^; or by the interaction of electrons with the tail of the plasma wave drive pulse^22–25^.

The betatron oscillation can also be tuned by manipulation of the plasma density. This can be done in several ways, e.g. by using a tilted shock front in the acceleration phase^26^, an axially modulated plasma density^27^, off-axis laser alignment to a capillary plasma waveguide^28^, transverse density gradient^29,30^, or tailoring the dynamics of the nonlinear plasma wave in a way that electrons find themselves behind its first period (the bubble) for a certain period of time, where their oscillations are amplified due to the opposite polarity of transverse fields^31^. Also, injection of matter by irradiating solid micro-droplets^32^ or nanoparticles^33^ may provide enhancement of the generated betatron X-ray intensity.

The conversion efficiency from laser-light to X-ray can be increased by using a hybrid scheme, which combines a low-density laser-driven plasma accelerator with a high-density beam-driven plasma radiator^34^. Increase of betatron light by localized injection of a group of electrons in the shape of an annulus was also reported^35^. The X-ray flux can also be increased due to shortening of the betatron oscillation wavelength during the natural longitudinal expansion of bubble^36^.

**Figure 1:**
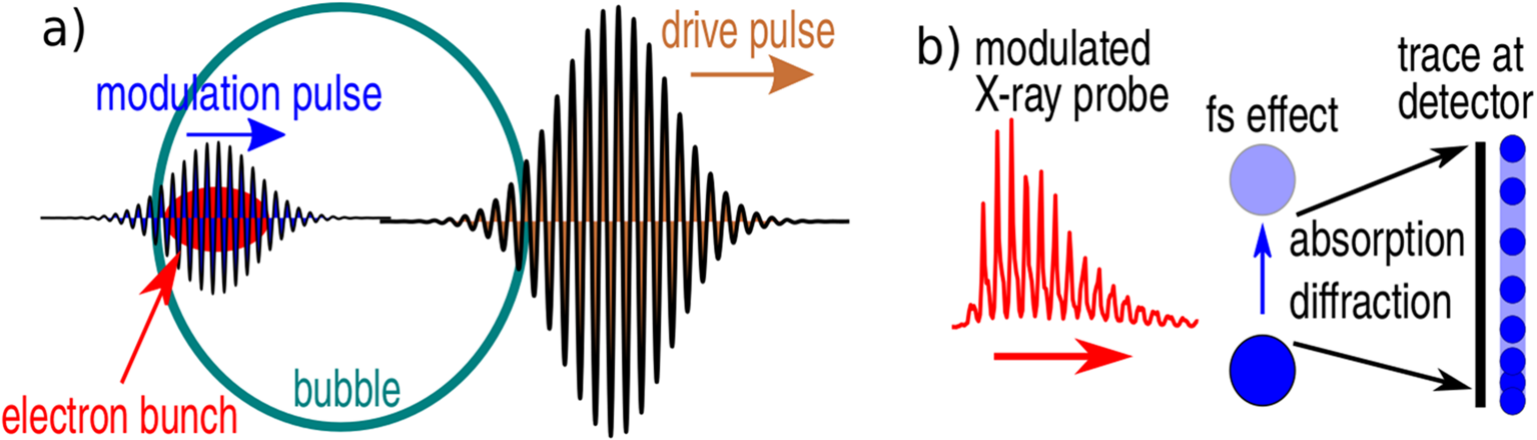
Schematics of the proposed setup and the application configuration. (**a**) A moderately high-intensity laser pulse creates a plasma cavity free of electrons (bubble). An electron bunch is injected in the rear part of the bubble, along with a weaker modulation pulse, with a delay that is such that it propagates with the electron bunch. (**b**) Illustration of stroboscropic measurement of fast processes using a modulated X-ray probe.

In this paper, we propose an experimental setup where, in addition to an enhancement of the betatron radiation flux, a train of sub-femtosecond X-ray pulses is generated. It is achieved by separation of the electron bunch accelerated in the laser wakefield into a train of equidistant sub-bunches by a delayed modulation laser pulse, see Fig. 1a) for a schematic of the proposed setup. The separation interval between the pulses corresponds to half of the modulation pulse wavelength and each pulse in the train is even shorter.

Generation of electron bunch trains has been studied previously. They originate either from conventional radiofrequency accelerators^37–39^, from laser wakefield accelerators employing self-injection controlled by driver pulse shaping^40^ or optical injection by crossing two wakefields^41^, or from plasma wakefield accelerator injected due to the bubble length oscillation on the density downramp^42^. The advantage of the scheme described in this paper over the aforementioned ones is that the electron bunching is well controlled by the modulator on the sub-micron scale. Thus, the emitted signal comprises of the train of X-ray pulses with an unprecedented repetition rate.

Pulse-trains composed of sub-femtosecond X-ray pulses can enhance the temporal resolution of sampling of ultrafast fundamental physical processes by an order of magnitude, whilst maintaining its other advantageous features such as a small source size of several microns enabling high-resolution images and a relatively small cost of the required laser systems compared to the large scale facilities such as synchrotrons or free electron lasers. A broadband X-ray pulse-train could sample physical processes occurring on femtosecond time-scales by e.g. X-ray absorption spectroscopy (XAS) or polychromatic (Laue) X-ray diffraction. In all cases, the image observed at the detector (typically a CCD camera) would be composed of a series of sharp and fuzzy regions. As the time-delay between the X-ray pulses in a train is set by the wavelength of the modulation pulse, the dynamics of the sampled process can be extracted from the configuration of the sharp region on the detected image. This approach is analogous to stroboscopic measurement of fast processes, see Fig. 1b) for a schematic illustration. In attosecond science, stroboscopic images have been already recorded^43^ with high harmonics emission^44^. Our source, despite being incoherent on its wavelength, provides higher photon energy which results in the increased penetrability through the investigated sample.

## Results

A driving laser-pulse of moderate intensity ($I \lesssim 10^{19}$ W cm$^{-2}$), linearly polarized in the *y*-direction, propagates in the longitudingal (*x*) direction in an underdense plasma (in practice, $n_e$ is in the order of $10^{18}$ cm$^{-3}$) and creates a moderately nonlinear plasma wave. Its first period, the so-called “bubble”, is an ion cavity free of electrons which are expelled by the strong ponderomotive force of the driving pulse. The electron bunch is located in the rear part of the bubble. It is injected transversely (*y*-direction), either by self-injection, or as is the case in this paper, by controlled injection on the density downramp. A weaker modulation pulse ($I \lesssim 10^{18}$ W cm$^{-2}$) with wavelength $\lambda _m$ is injected to follow the driving pulse. Its electric field, polarized in the *y*-direction, still dominates over the electrostatic transverse field of the bubble. The delay between the pulses is chosen in a way that its high-intensity part co-propagates with the electron bunch.

As the modulation pulse propagates within the bubble, its group velocity is approximately equal to the speed of light in vacuum $v_{g,m} \lessapprox c$. The average longitudinal velocity of an electron in the bunch is lower, due to the relativistic limitation caused by transverse betatron oscillations. The accelerated electrons oscillate transversely on a sine-like trajectory because they gained a considerable transverse momentum dominantly by the fields of the modulation pulse, but also by the injection process and by the electrostatic transverse fields of the bubble. Every periodic increase of their transverse velocity leads to a decrease of their longitudinal velocity. As a result, the modulation pulse steadily overtakes the electron bunch. Consequently, an electron from the bunch experiences the action of a periodically varying transverse component of the Lorentz force as it propagates backward with respect to the modulation pulse.

The transverse electron motion can be described by the equation of motion ${\text {d}}p_y/{\text {d}}t \approx q_e(1-\beta _x)E_{0,y,m}\cos (k_m\xi )$, where $q_e$ is electron charge, $E_{0,y,m}$ is the electric field amplitude of the modulation pulse, $k_m\xi$ is the phase of the modulation pulse, with $k_m=2\pi /\lambda _m$ being the modulation pulse wavenumber and $\xi = x - x_0 - v_{g,m}t$ the coordinate co-moving with the modulation pulse. Here, we assumed $|p_x| \gg |p_y|$, $p_x \gg m_ec$, and considered the modulation pulse as a plane wave, which is applicable in regions around the propagation axis, where its magnetic field is proportional to its electric field $B_z \approx E_y/c$. Thus, the electrons flow backward with respect to the modulation pulse and due to the phase dependence of the transverse force, they are periodically pushed in the $\pm y-$direction. This effect itself leads to enhancement of the betatron radiation emission in comparison with a standard case without the modulation pulse.

From the positions where $\cos (k_m\xi ) =0$, the absolute value transverse momentum of the electrons decreases and the longitudinal momentum grows; the latter one is largest at the turning points of their trajectory where $p_y=0$. Thus, the turning points related to the modulation pulse phase are the same for all electrons of the bunch. Large longitudinal momenta together with low transverse momenta result in a clustering of the bunch electrons in the nests co-moving with the modulation pulse. Alternatively stated: the original electron bunch is microbunched. As the betatron radiation is mainly emitted at the turning points of the electron trajectories, its temporal profile is composed of intensity peaks separated by $\lambda _m/2c$, i.e. a train of X-ray pulses is emitted and the delay between the pulses is adjustable by choosing $\lambda _m$.

The effect of microbunching can be understood as a forced betatron resonance. Contrary to previous cases with the modulation by the tail of the plasma wave drive pulse^23,25^, where the electron beam experiences a long acceleration period before it catches the laser pulse which resulting in limited controllability of the X-ray source, we reach the betatron resonance immediately from the moment of injection.

### Numerical simulation

The process of michrobunching and its fingerprint on the betatron radiation signal is studied by means of 2D particle-in-cell (PIC) simulations and their post-processing. A bubble regime configuration with modest laser parameters is chosen for the demonstration of the process. The parameters used in the simulation are the following: plasma electron density $n_0 = 2.5\times 10^{18}$ cm$^{-3}$, driver laser wavelength $\lambda _d = 0.8$ $\upmu$m, waist size (radius at 1/e^2^ of maximum intensity) $w_0 = 10$ $\upmu$m, pulse length (FWHM of intensity) $\tau =20$ fs, and normalized driver laser intensity $a_{0,d} = eE_{0,d}/m_ec \omega _0=1.8$ which corresponds to intensity $I=6.9\times 10^{18}\;{\mathrm{W}}\;{\mathrm{cm}}^{-2}$. Its focal spot is located at $x_{f,m}=110$ $\upmu$m. The modulation pulse has the same fundamental parameters with the exception of normalized intensity, which is $a_{0,m}=0.2$, and wavelength $\lambda _m = \lambda _d/3$ corresponding to intensity $7.7\times 10^{17}\;{\mathrm{W}}\;{\mathrm{cm}}^{-2}$. It is delayed by 58 fs and its focal spot is located at $x_{f,m}=410$ $\upmu$m. Both pulses are linearly polarized in the $y-$direction.

Self-injection of electrons in the plasma wakefield does not occur with these parameters if the plasma density is constant. Instead, a plasma density profile is chosen so that controlled injection occurs. In the simulations, the density profile is set in the following way. A 10 $\upmu$m long vacuum is located at the left edge of the simulation box, then a 50 $\upmu$m linear density up-ramp follows until the electron density reaches $2n_e$. Nevertheless, the nature of the presented injection scheme does not depend on the plasma-edge density ramp. Afterwards, a 35 $\upmu$m long density plateau follows; then the density linearly drops to $n_e$ over a distance of 25 $\upmu$m. On this down-ramp, the controlled injection occurs^45^. The PIC simulations were performed with the epoch code, see the Methods section for details.

**Figure 2:**
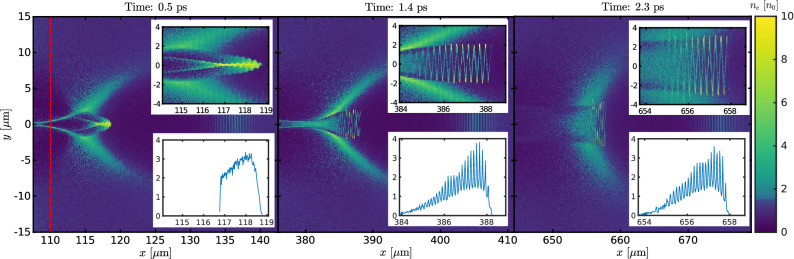
Plasma bubble evolution and electron microbunching. Snapshots of the electron density at the injection time ($0.5 \;{\mathrm{ps}}$ left panel) and during the acceleration process ($1.4 \;{\mathrm{ps}}$ and $2.3\;{\mathrm{ps}}$, centre and right panels, respectively). The red line in the left panel represents the end of the initial density down-ramp. Only a central part of the simulation box is shown. The upper insets show a zoom of the bunch structure, the bottom insets show a projection of the trapped particles density on the *x*-axis.

The snapshots of the electron density during the injection and acceleration process are shown in Fig. 2. The density profile in the panel corresponding to the injection time ($t=0.5$ ps) suggests that the electron bunch is microbunched immediately after the injection. In later times (1.4 ps and 2.3 ps of simulation), the snake-like structure of the bunch is pronounced.

**Figure 3:**
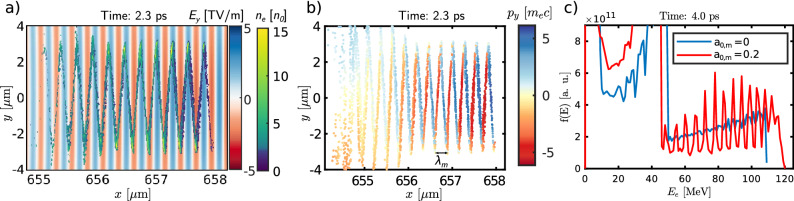
Electron bunch structure and energy spectrum. (**a**) Electron density of the trapped electrons (plotted the simulation cells where average kinetic energy of electrons is higher than 10 MeV) and the transverse electric field at $t=2.3\;{\mathrm{ps}}$. (**b**) Transverse momentum of the trapped electrons. (**c**) Electron energy spectra at $t=4.0\;{\mathrm{ps}}$ for the cases with and without modulation pulse present.

The detailed view of the electron bunch structure at 2.3 ps is shown in Fig. 3a), together with the transverse electric field. Apparently, the electric field of the modulation pulse dominates over the electrostatic field of the bubble in the region around the axis where the electron bunch is located. The bunch itself has a sawtooth-shape. The distance between the $x-$coordinates of the turning points is $\lambda _{m}/2$. The peak values of the electron density are located in these turning points.

Figure 3b) shows the positions and transverse momenta of the accelerated electrons. The positions between the peaks of the density bunch profile and the dominant direction of the transverse component of the electron momentum confirm that the electrons propagate backwards in the frame co-moving with the modulation pulse. These findings can be interpreted as the electron bunch as a whole performs snake-like motion in the direction of $-\xi$. This means that the modulation pulse effectively induces the microbunching of injected electrons and the distance between single microbunches is $\lambda _{m}/2$ in the longitudinal direction.

The electrons perform betatron oscillations, however, in contrast to standard betatron motion in the case without the modulation pulse, the oscillations are driven dominantly by the modulation pulse. Thus, crucially, the turning points are the same for all of the trapped electrons. In other words, the electron bunch is effectively separated into several equidistant microbunches that are continuously radiating. As a consequence, the observer will receive a modulated betatron radiation signal, comprising of peaks arriving every $\lambda _m/2c$, as will be shown later.

The electron energy spectrum in time of 4.0 ps just before the structure begins to dephase is shown in Fig. 3c); blue and red lines show the cases without and with the modulation pulse, respectively. The spectra comprise a clear peak which corresponds to the electrons accelerated in the first period of the plasma wave due to the controlled injection. Although, the relative energy spread is rather high. However, for the purpose of betatron radiation generation the energy spread is not a determining factor. The presence of the modulator leads to further electron energy gain compared to the reference case: the electrons receive the energy stored in the modulator by direct laser acceleration^46,47^. The estimated accelerated charge (electron energy higher than 25 MeV) is about 4 to 8 pC in both cases. There are about 1.3% less electrons trapped when the modulator is present.

### Betatron radiation spectrogram

**Figure 4:**
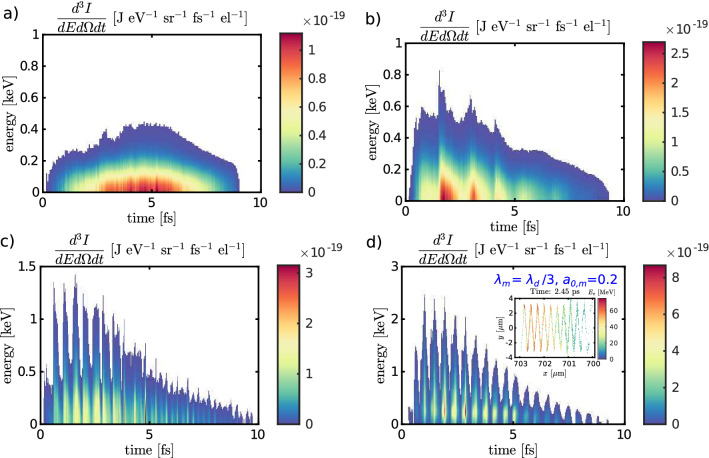
Spectrograms of the betatron radiation emitted by the electrons. Temporal and energy profiles are shown for a reference case without a modulator and for three different modulator pulse cases. The signal close to $t = 0$ corresponds to the front of the bunch and arrives first at the detector. The inset in the panel (**d**) shows the electron energy distribution within the bunch. It displays a matrix of the average electron energy in cell; only the cells with average energy over 10 MeV are shown. Both temporal and energy profile of emitted X-rays are correlated with the inner structure of the bunch. Note that the *x*-axis is reversed.

Figure 4 shows the spectrograms, i.e. both temporal and energy profiles of the betatron radiation, with and without the modulation pulse; for details see the Methods section. Four different cases are presented: a) the case when the modulator is not present, (b) with $\lambda _m = \lambda _d$ and $a_{0,m}=0.6$, (c) with $\lambda _m = \lambda _d/3$ and $a_{0,m}=0.1$, and (d) with $\lambda _m = \lambda _d/3$ and $a_{0,m}=0.2$. The results presented in Figs. 2 and 3 correspond to case (d).

All the signals are approximately 10 fs long, corresponding to a bunch length of $\approx$ 3.5 $\upmu$m shown in Fig. 3. Nevertheless, while the signal is continuous in the case without the modulator (Fig. 4a), the modulated signals (Fig. 4b–d) exhibit trains of ultrashort pulses. Moreover, the spectrograms show that the betatron radiation critical energy is also modulated in time. In average, the energy of radiation is considerably higher when the modulator is present. The inset in panel (d) confirms the correlation between the energy distribution of electrons within the bunch and the temporal and energy profile of emitted X-rays.

**Figure 5:**
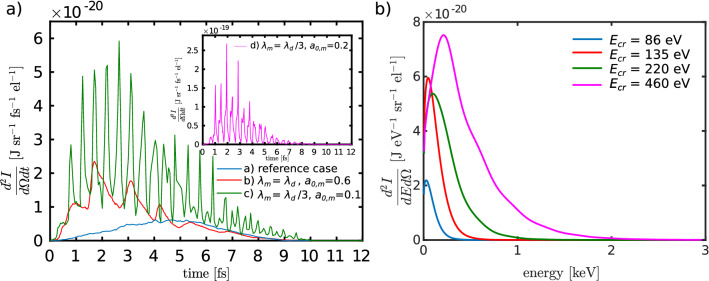
Energy spectra for the emitted betatron radiation. (**a**) Temporal profile of the betatron radiation for a reference case without a modulator and for three different modulator pulse cases. The inset, corresponding to the case $\lambda _m = \lambda _d/3$ and $a_{0,m}=0.2$ is an order of magnitude more intense than the other cases. (**b**) On-axis time-integrated energy spectra of emitted X-rays and the critical energy of the emitted signal for the four cases (**a**) no modulator, (**b**) $\lambda _m = \lambda _d$ and $a_{0,m}=0.6$, (**c**) $\lambda _m = \lambda _d/3$ and $a_{0,m}=0.1$, and (**d**) $\lambda _m = \lambda _d/3$ and $a_{0,m}=0.2.$

Figure 5 shows the temporal profiles of betatron radiation. Whereas the blue curve belonging to reference case (a) does not vary significantly, the other three curves (b–d) show several clear peaks. The red curve represents the case (b); three dominant peaks are present. The peak-to-peak distances is between the first and the second and the second and the third dominant peaks are 1.35 fs and 1.29 fs, respectively. This is in good agreement with the theoretically expected value $\lambda _m/2c=1.\overline{3}$ fs. The green curve corresponds to case (c). The signal comprises of more than thirteen clear peaks. The peak-to-peak distance is (0.46 ± 0.02) fs (estimated by Fourier transform of signal) and is in good agreement with the expected value of $\lambda _m/2c=0.\overline{4}$ fs. Such a feature can be interpreted as a betatron radiation pulse train coherence with respect to the modulation pulse.

The radiation peaks themselves are even shorter, the FWHM of the brightest one at 2.65 fs is 140 as. There is a considerable continuous background, the pulsed signal to noise ratio is about 5:1. This ratio could be significantly improved by employing a transmission filter which effectively cuts the low energy parts of the spectra.

The inset of Fig. 5a contains the last case (d). The signal is an order of a magnitude more intense than the other cases. It is bunched, with a signal-to-noise ratio of better than 20:1. Again, Fourier transform of this signal shows that the fundamental period is (0.45 ± 0.01) fs, and the FWHM of the brightest peak at 1.92 fs is 100 as.

The number of electrons within the bunch differs by less than 3.5% between all four compared cases.The estimated total energy within the pulse train is 0.10 nJ in case (a). It increases greatly when the modulator in present: it is 0.45 nJ, 0.65 nJ, and 2.2 nJ in cases (b–d), respectively. The increase is caused partly by the higher energy of the electrons and partly by the higher amplitude of betatron oscillations.

Finally, the time-integrated energy spectra on axis for all the cases (a–d) are shown in Fig. 5b, including information about the critical energy of the emitted signal in all cases. The critical energy of the case (d) is 5.3$\times$ higher than in the reference case (a).

## Discussion

We propose a method for producing a train of ultrashort X-ray pulses by modifying the standard laser wakefield accelerator setup delivering betatron radiation. This is accomplished by adding a delayed modulation laser pulse to follow the plasma wave in the region where the electron bunch is injected. As a result, the betatron oscillations of the accelerated electrons are driven dominantly by the fields of the modulation pulse and not by electrostatic fields of the bubble. The turning points of the betatron trajectories are the same for all accelerated electrons and the electrons cluster there.

In other words, the electron bunch is microbunched and the longitudinal distance between the single bunches is half of the modulation pulse wavelength $\lambda _m$. This property is imprinted on the temporal profile of the emitted X-rays. Thus the betatron radiation signal is composed of a train of pulses separated by a factor of $\lambda _m/2c$, which is 440 as when third harmonics of a standard Ti:sapphire laser pulse is used as the modulator. Moreover, the energy and intensity of the emitted X-rays are also enhanced. The resulting X-ray source could enable observation of temporal evolution of ultrafast phenomena on the time scale of hundreds of attoseconds.

The process of electron microbunching was further tested in a relatively broad parameter space. The scheme works in the densities $1.8\times 10^{18}$ cm$^{-3}$–$6\times 10^{18}$ cm$^{-3}$. The sharpest microbunching occurs in lower densities, as higher density leads to the lower plasma wave phase velocity causing the structure decay due to dephasing. The results that are presented throughout the paper are given after 3.5 ps of acceleration time ($t=4.0$ ps). This corresponds to the time when the spectrogram is the sharpest for the main demonstration case (d).

Furthermore, the intensity of the modulator pulse was varied and the stability of the scheme was confirmed. Generally, it can be stated that the scheme works in the parameter range where the modulator pulse field is higher than the transverse electrostatic field of the bubble, but low enough to avoid the significant disruption of the plasma wave. Approximately, this correspond to the normalized modulator pulse intensity of $a_{0,m} \in [0.05,0.4]$. Within this parameter region, the more intense modulator leads to the better bunching.

We close with two example applications where the suggested technique has the potential to drive forward development. Betatron radiation has already been used in laboratory astrophysics, when warm dense matter (WDM) samples were investigated employing XAS^48^. It takes advantage of the broadband photon spectrum in the keV region, where most elements’ absorption edges are located. The time-resolved XAS technique pushes its limits from hundreds of picoseconds by synchrotrons or streak cameras to femtoseconds by a betatron source. The presented technique provides an improvement of the XAS time resolution by an order of magnitude.

Broadband synchrotron X-ray pulses are used also in solid state physics for polychromatic (Laue) X-ray diffraction^49,50^, where the different energies are diffracted in different angles. In the standard monochromatic X-ray diffraction, time resolved synchrotron pulses are used to sample the nonlinear lattice dynamics, in particular, to determine the crystal structure of solids and its evolution^51,52^. The pulse train produced by our scheme allows the development of sub-femtosecond time resolved polychromatic X-ray diffraction.

## Methods

2D PIC simulations were performed with the EPOCH^53^ code. The simulations were run in the moving simulation box with dimensions 80 $\upmu$m $\times$ 40 $\upmu$m. The grid resolution was 90 and 12 cells per $\lambda _d$ in the longitudinal and transverse directions, respectively. Initially, two electron macroparticles were placed in every cell. The plasma is represented as an electron gas; the ions were considered as a homogeneous static background. In total, approximately $2.2\times 10^8$ macroparticles were simulated.

The temporal profile of betatron radiation was calculated using the method based on the Fourier transform of the emitted signal which can be determined by using trajectories of the trapped electrons^54^. It takes advantage of the fact that each electron performs betatron motion in the wiggler regime and the emitted signal is composed of a series of sharp peaks radiated at the turning points of the electron trajectories separated by relatively long intervals of silence. Thus, it is possible to store the times when the single peaks of all the tracked electrons were emitted and construct the betatron radiation spectrogram from that. This method is applicable even for the discussed case of X-ray emission by microbunched electrons, because the level of microbunching does not suffice to emit coherent electromagnetic radiation more energetic than ultraviolet. 20 000 of the tracked electron macroparticles were processed in each case.
